# Comment on “The Effects of Hemodialysis on Tear Osmolarity”

**DOI:** 10.1155/2016/6890685

**Published:** 2016-03-16

**Authors:** Onder Ayyildiz, Gokhan Ozge

**Affiliations:** Department of Ophthalmology, GATA Medical School, 06010 Ankara, Turkey

We congratulate Taskapili et al. for their study entitled “The Effects of Hemodialysis on Tear Osmolarity” [1]. The authors investigated the effects of hemodialysis (HD) on tear osmolarity (TO) and evaluated the correlation between blood biochemical tests and TO in patients with end stage renal disease (ESRD). In this study, they evaluated TO of patients with ESRD one minute before the beginning of HD and 30 minutes after the end of HD. The authors established that HD effectively decreases TO to normal values and corrects the volume and composition of the ocular fluid transiently. They observed tear hyperosmolarity before HD and a significant reduction to normal levels after HD.

We have read the paper with great interest. We noticed that the authors did not mention the time of HD start in the day. It was reported that there was a diurnal variation of TO for normal subjects, which significantly changes upon awakening and remains relatively constant throughout most of the day [2]. In this study it was stated that the tear film was hypoosmotic upon awakening compared with baseline and TO changed quickest within the first 10 minutes and increased to baseline levels within the first 40 minutes [2]. TO decreased slowly thereafter until the 8-hour visit, followed by a relatively hyperosmotic trend toward the end of the day [2]. We think that detection of TO would be performed at the same time of the day regarding the duration of HD which may avoid the bias of the methodology.
